# Occurrence of *Bifidobacteriaceae* in human hypochlorhydria stomach

**DOI:** 10.3402/mehd.v25.21379

**Published:** 2014-01-09

**Authors:** Paola Mattarelli, Giovanni Brandi, Carlo Calabrese, Fabio Fornari, Gian Maria Prati, Bruno Biavati, Barbara Sgorbati

**Affiliations:** 1 Department of Agricultural Sciences, Bologna University, Bologna, Italy; 2 Institute of Haematology and Medical Oncology, Bologna, Italy; 3 Department Clinical Medicine, S. Orsola-Malpighi Hospital, Bologna University, Bologna, Italy; 4 Gastroenterology Department, Piacenza Hospital, Piacenza, Italy

**Keywords:** Bifidobacteriaceae, hypochlorhydria stomach, Actinomycetales, omeprazole-treated gastritis, autoimmune gastritis

## Abstract

**Background:**

The human stomach, when healthy, is not a suitable host for microorganisms, but in pathological conditions such as gastritis, when gastric acid secretion is impaired, microbial overgrowth can be observed. Apart from *Helicobacter pylori*, the composition of microbiota, resident or exogenously introduced during neutral/high pH conditions, has not been investigated thoroughly. Thus, it is possible that *Bifidobacteriaceae*, important autochthonous and beneficial bacteria of human gastrointestinal microbiota, could over-colonize the stomach of hypochlorhydria patients suffering from autoimmune atrophic gastritis (AAG) or omeprazole-treated (OME) gastritis. This prompted us to characterize the *Bifidobacteriaceae* in such patients’ gastric microbiota and to study its abnormal colonization.

**Methods:**

Samples of gastric juices, and antrum and corpus mucosa from 23 hypochlorhydria patients (13 AAG and 10 OME) and from 10 control volunteers with base-line normochlorhydria, were cultivated in Brain Heart Infusion (BHI) and selective *Bifidobacterium*-Tryptone-Phytone-Yeast extract (Bif-TPY) media. The isolates were characterized by the fructose-6-phosphate phosphoketolase (F6PPK) test, electrophoresis of cellular proteins, the fermentation test, guanine-cytosine% DNA content, and DNA–DNA hybridization. Negative F6PPK isolates were characterized by order-specific polymerase chain reaction (PCR).

**Results:**

A total of 125 isolates, assigned to the *Bifidobacteriaceae* family on the basis of their morphology, were obtained from AAG and OME patients, but not from normal subjects. Of these isolates, 55 were assigned to the *Bifidobacteriaceae* family on the basis of their fructose-6-phosphoketolase (PPK) activity, PPK being the key taxonomic enzyme of this family. The remaining 70 isolates, which were PPK-negative, were attributed to the *Actinomycetales* order following specific primer PCR analysis. We observed a significantly higher abundance of *Bifidobacteriaceae* (*Bifidobacterium dentium*, *Scardovia inopinata*, and *Parascardovia denticolens*) in OME group than the AAG group. Furthermore, the *Actinomycetales* distribution was homogeneous for both hypochlorhydria patient groups.

**Conclusions:**

This study suggests that the *Bifidobacteriaceae* species, typically found in the oral cavity, readily colonizes the hypochlorhydria stomach of OME patients. The clinical relevance and the mechanism underlying this *Bifidobacteriaceae* presence in OME gastritis requires further functional studies.

*Bifidobacteriaceae* are indigenous components of human and animal gastrointestinal microbiota and are routinely isolated from the human gastrointestinal tract, especially the colon; they are the first and most dominant gut inhabitants in early human life. *Bifidobacteriaceae* are also present in the oral cavity and the vagina (1). Until now, the stomach has been considered an inhospitable environment for microorganisms because of its gastric acidity (2). However, recent molecular techniques have revealed that the normal acidic stomach may be the habitat of a distinct microbial ecosystem, the most common bacterial phyla being *Proteobacteria*, *Firmicutes*, *Bacteroidetes*, *Actinobacteria*, and *Fusobacteria*, and the most abundant genera *Helicobacter*, *Streptococcus*, and *Prevotella* (3). It is known that impaired gastric acid secretion caused by chronic atrophic gastritis, the prolonged use of histamine-2 receptor antagonists or proton pump inhibitors, can be associated with bacterial overgrowth in the stomach (4). Apart from *Helicobacter pylori*, the composition of the microbiota, resident or exogenously introduced during neutral/high pH conditions, has not been thoroughly investigated. A recent study of Dicksved et al. (5) pointed out that acid reducing drug therapy, corpus atrophy, and gastric cancer can lead to the microbiota overgrowth being dominated by different species of the genera *Streptococcus*, *Lactobacillus*, *Veillonella*, and *Prevotella*. Considering that *Bifidobacteriaceae* are components of oral microbiota (6, 7), and that in conditions of reduced gastric acid secretion bacterial overgrowth appears related to upstream colonization in the alimentary tract, it is surprising that so many of the recently conducted, large-scale studies (3, 5, 8, 9) (but see also (10)) failed to detect *Bifidobacteriaceae* in the hypochlorhydria stomach microbiota.

The aim of this study was to evaluate, using culture dependent methods, the *Bifidobacteriaceae* distribution in the hypochlorhydria stomach of patients with either autoimmune atrophic gastritis (AAG) or omeprazole-treated (OME) gastritis.

## Materials and methods

### Patients

A total of 33 patients (mean age 48.15±14.71, range 20–71, 15 men) underwent upper gastrointestinal endoscopy at around 8.00 a.m. Of these, 23 patients had the hypochlorhydria condition: 10 with AAG (mean age 43.70±19.14 year, range 20–71 year, four men) and 13 treated with OME (20 mg/day) for peptic disease (mean age 52.85±9.02 year, range 42–70 year, six men). The control group (mean age 46.5±15.41, range 20–71 year, five men) consisted of 10 volunteers with base-line normochlorhydria (fasting gastric pH <4). All of the subjects gave their informed written consent to the study, which involved upper gastrointestinal endoscopy and biopsy procedures.

### Juice and gastric biopsies

A sample of fasting gastric juices (5–10 ml) was aspirated at endoscopy using a sterile Teflon cannula inserted into the biopsy channel of the endoscope. The closed cannula was opened only after it reaches the gastric lumen. For the biopsies, six specimens were taken from the gastric antrum (2 cm proximal to the pylorus) and six from the corpus (10 cm below the gastroesophageal junction along the greater curvature) using two different, sterile biopsy forceps (Olympus FB 24Q-1, Tokyo, Japan). Before each test, the endoscopes (Olympus GIF 130, Tokyo, Japan) were disinfected with glutaraldehyde 2% and the biopsy channel with 70% ethanol; then rinsed with sterile water. Gastric juice and four biopsies per area were taken to assess bacterial growth. The gastric juice pH was measured using a pH-meter (HANNA-8521) with the microelectrode HI 2031B.

### Bacterial isolation

The gastric juice samples and biopsy specimens were immediately processed for bacteriological evaluation. The biopsy material was washed with sterile saline solution, first by gentle hand shaking then harshly by vortex to avoid bacterial contamination from the gastric fluid. The biopsy material was then weighed, homogenized, and diluted in saline solution using a sterile procedure. Each 100 µl aliquot of serial diluted homogenate and gastric juice (10^−2^-10^−8^) was plated on Brain Heart Infusion (BHI) agar for anaerobic bacterial growth, and on selective *Bifidobacterium*-Tryptone-Phytone-Yeast extract (Bif-TPY) medium (1) containing 5% propionic acid (pH 5.0) (11) for bifidobacterial growth. The plates were then incubated anaerobically at 37°C for 5 days.

Colonies containing cells with bifidobacterial morphology characteristics were picked and subcultured in 0.5% agar TPY stab anaerobically, at 37°C for 24 h. The isolates were then subcultured in TPY liquid medium with the same incubation conditions for the identification assays. The cells, after centrifugation and addition of cryoprotective medium (skim milk 20%, lactose 0.3%, yeast extract 0.3%), were maintained both frozen at –135°C and freeze-dried in the Bologna University Scardovi Collection of *Bifidobacterium* (BUSCOB).

### *Bifidobacteriaceae* species identification and characterization

Morphological features and growth type observations were performed according to Crociani et al. (12). The presence of fructose-6-phosphate phosphoketolase (F6PPK) was determined as described by Biavati and Mattarelli (1). Species identification was carried out by means of fermentation tests, polyacrylamide gel electrophoresis (PAGE) of soluble proteins, and DNA–DNA homology. Fermentation tests were performed in TPY liquid medium as described by Crociani et al. (13). Table 1 shows the 51 tested substrates (simple and complex carbohydrates, polyalcohols, mucins, and gums). The PAGE of soluble proteins, DNA base compositions (G + C% contents), and DNA–DNA homology were carried out as previously described by, respectively, Biavati et al. (14), Crociani et al. (12) and Biavati and Mattarelli (1). Nitrate reduction was determined in a modified TPY medium containing 0.1% wt/vol of KNO_3_ and glucose, evidenced by the sulfanilic-naphthylamine reagent (15).

**Table 1:** Fermentative characteristics of the *Bifidobacteriaceae* strains isolated from the hypochlorhydria stomach^a^

	Species				
	
Substrate	*P. denticolens*	*S. inopinata*	*B. dentium*	*B. infantis-longum*	Unidentified strain
	34^b^				
	14	5	1	1	
Amylopectin	+	+	+	+	−
Amylose	+	+	−	−	−
l-Arabinose	+ (3)^c^	−	+	−	+
Cellobiose	+ (1)	− (1)	+	+	+
Dextran	+ (2)	+	− (1)	−	−
Dextrin	+	+	+	+	−
Galactose	+	+(1)	+	+	+
Glycerol	−	− (1)	−	−	−
d-Glucosamine	+	−	−	−	−
Gum guar	−	−	+	−	−
Gum locust bean	−	−	+	−	−
Inulin	+	− (1)	−	+	−
Mannitol	−	−	+	−	−
Mannose	−	−	+	−	−
Melezitose	+ (4)	+ (2)	+	−	+
Raffinose	+ (3)	+	+	+	+
Salicin	+ (1)	− (7)	+	+	+
Trehalose	−	−	+	−	−
d-Xylose	− (4)	+ (1)	+	+	+

+, positive reaction; −, negative reaction.

a All strains fermented glucose, fructose, lactose, melibiose, maltose, ribose, sucrose, starch. None of the strains fermented alginate, arabinogalactan, bovine submaxillary mucin, chondroitin sulfate, d-fucose, l-fucose, d-galactosamine, alpha-d-galacturonate, gluconate, d-glucuronate, gum arabic, gum ghatti, gum karaya and gum tragacanth, l-hyaluronate, lactate, laminarin, ovomucoid, pectin, polygalacturonate, porcine gastric mucin, l-rhamnose, sorbitol, xylan.

b Number of strains tested.

c In parenthesis, the number of strains with the opposite reaction.

### *Actinomycetales* group identification

Phosphoketolase negative isolates were tested by primer-specific polymerase chain reaction (PCR), to identify the *Actinomycetales* order; DNA was extracted according to Rossi et al. (16). A pair of universal primers designed for the order of *Actinomycetales* and the PCR procedure, described by Xia and Baumgartner (17), was used to identify the isolates. The PCR-positive controls were done with DSM 43327^T^ *Actinomyces viscosus*, and the negative ones with double-distilled water.

### Statistical analysis

Parametric results were reported as means with SD. A statistical comparison was made between the detection rate of *Bifidobacteriaceae* and *Actinomycetales* groups and stomach hypochlorhydria in OME and AAG subjects, performed using Fisher's exact test. *p*<0.05 was considered significant.

## Results

Table 2 shows the demographic and clinical characteristics of the 33 subjects classified according to pathology and gastric pH.

**Table 2:** Demographic and clinical characteristics of the 33 subjects studied

	AAG	OME	Acid control
Number of subjects	10	13	10
Sex (M/F)	4/6	6/7	5/5
Age (mean years±SD)	43.7±19.1	52.9±9.0	46.5±15.4
pH juice (mean±SD)	7.12±0.8	7.41±0.5	0.9±0.6
Patient with *Bifidobacteriaceae* only	1	2	0
Patients with *Actinomycetales* only	8	6	0
Patients with both *Bifidobacteriaceae* and *Actinomycetales*	1	7	0

### pH and anaerobic microbial growth

The gastric pH was significantly lower in the acid controls (0.90±0.6) than in the AAG and OME groups (7.42±0.5 and 7.12±0.8, respectively) (*p*<0.01), while the OME and AAG groups showed no significant difference (*p*>0.05) (Table 2). All of the examined subjects showed a significant positive correlation between gastric pH and total bacterial count (*r* ^2^=0.89; *p < 0.01*), higher microbial counts being evident in the high pH stomach (Table 3).

**Table 3:** Anaerobic microorganisms grown on BHI in gastric juice and mucosa of the stomach^a^

	Gastric juice^b^	Antrum^b^	Corpus^b^
AAG (10)	6.94±0.3	6.51±0.3	6.86±0.2
OME (13)	5.93±0.3	5.43±0.4	5.15±0.5

a In the acid control group, there is no bacterial growth in any sample (gastric juice and antrum and corpus mucosa)

b Mean bacterial count expressed as log10 values of CFU/g or ml.

Samples from antrum and corpus biopsies, and from gastric juice, were analyzed. Anaerobe growth in BHI broth was detected in all the samples from the AAG and OME subjects, but not from the acid controls (Table 3).

### *Bifidobacteriaceae* and *Actinomycetales* strains in the hypochlorhydria stomach

**Fig. 1:**
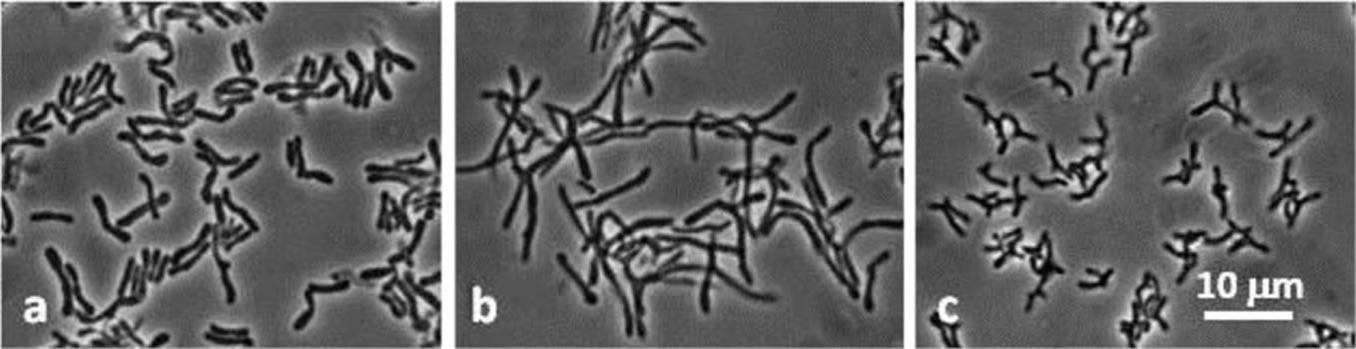
Morphology of *Actinomycetales* isolates: a, BR183; b, BR193; c, BR431. Phase-contrast microphotographs.

Isolates were obtained from Bif-TPY and BHI agar plates (45 and 80 colonies, respectively) containing cells with bifidobacterial morphological characteristics. The test for F6PPK, the key enzyme of the glucose catabolic pathway in the *Bifidobacteriaceae* family, was positive in all 45 isolates from selective Bif-TPY medium, and in 10 of the 80 isolates from BHI. Surprisingly, despite their typical bifidobacterial morphology (Fig. 1), the remaining 70 isolates from BHI (all F6PPK negative) did not belong to *Bifidobacteriaceae*. Thus, based on PCR analysis, and using a specific primer for *Actinomycetales*, they were presumptively assigned to the *Actinomycetales* order.

To sum up, 55 *Bifidobacteriaceae* and 70 *Actinomycetales* isolates were obtained from the 23 hypochlorhydria subjects. The 55 *Bifidobacteriaceae* isolates came from 2 of the 10 samples of AAG subjects, and from 7 of the 13 OME subjects, while the 70 *Actinomycetales* isolates came from 9 of the 10 AAG subjects and 11 of the 13 OME subjects (Table 2). For both AAG and OME patients, there was no correlation between the presence of *Bifidobacteriaceae* and *Actinomycetales* with respect to sex and patient age. However, there was a significant correlation between *Actinomycetales* presence and the AAG and OME patients (*p*<0.01), while the *Bifidobacteriaceae* presence correlated significantly only with the OME patients (*p*<0.01).

### Bifidobacteriaceae identification

**Fig. 2:**
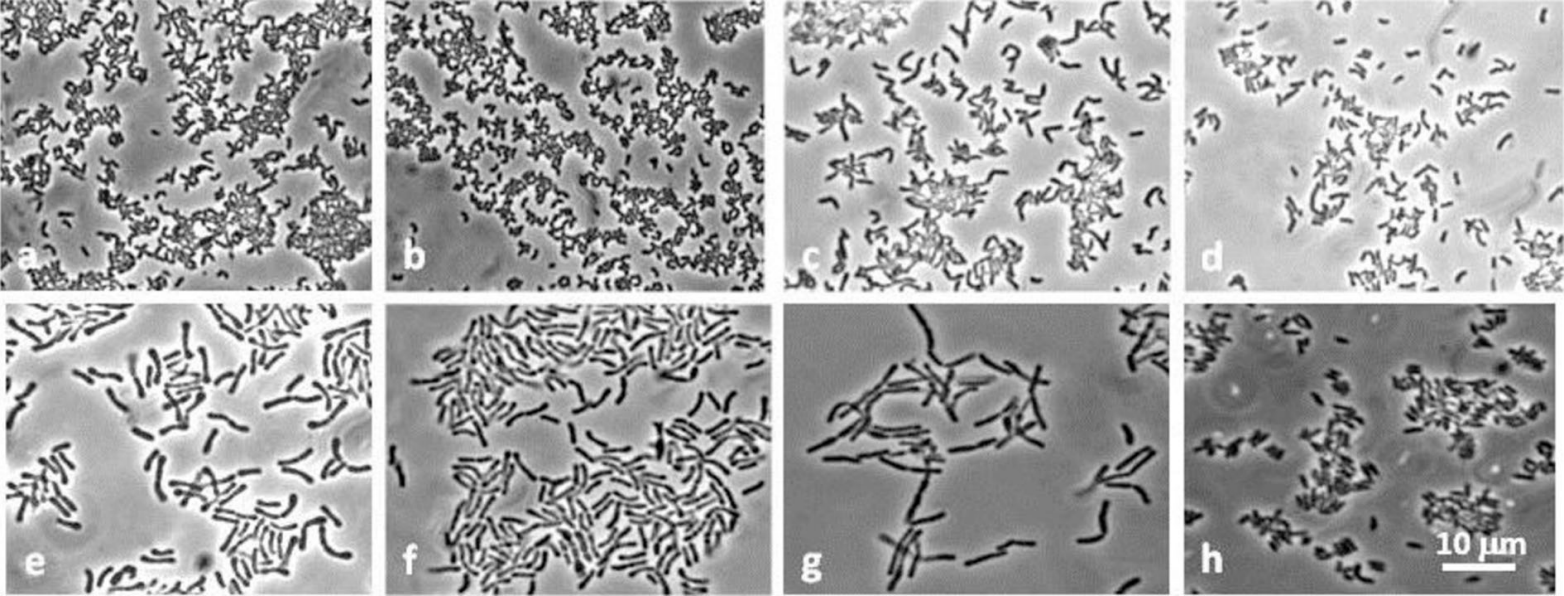
Morphology of *Bifidobacteriaceae* isolates. *S. inopinata*: a, BR134; b, BR203. *P. denticolens*: c, BR278; d, BR281. *B. dentium*: e, BR317; f, BR318. *B. infantis-longum*: g, BR184. Unidentified *Bifidobacteriaceae*: h, BR191.

The 55 *Bifidobacteriaceae* isolates underwent phenotype and genotype analyses that took into consideration cell morphology (Fig. 2), electrophoresis PAGE, fermentation, DNA G + C% contents and DNA–DNA homology analysis. All the utilized techniques consistently resulted in the same isolate grouping. These 55 *Bifidobacteriaceae* isolates were found to belong to three prevalent species, *B. dentium* (five isolates), *Scardovia inopinata* (14 isolates), *Parascardovia denticolens* (34 isolates) (Table 4), while one OME juice isolate belonged to the *B. infantis-longum* group, its homology value being 68 and 71% compared to the *B. longum* subsp. *longum* and *B. longum* subsp. *infantis* species, respectively. One OME antrum isolate, BR 191, failed to hybridize with any of the *Bifidobacteriaceae* species described to date (data not shown), so further analyses are needed to verify its belonging to a new species. Its DNA G + C content was 43%, one of the lowest in the *Bifidobacteriaceae* family; the intraspecific DNA–DNA homology range was 85–100%, whereas the interspecific was 5–15%.

**Table 4:** Demographic, clinical characteristics, and bacteria isolated in the hypochlorhydria stomach of the nine *Bifidobacteriaceae-*positive patients

Patients	Sex	Age	pH juice	Sample	Log10 CFU/g or ml	*Bifidobacteriaceae* species
AAG groups (2 of 10 patients)						
1	F	60	7.84	Juice	5.30	Sca.
				Corpus	4.30	Sca.
2	M	33	8.11	Juice	4.30	Par.
OME groups (7 of 13 patients)						
3	M	52	7.80	Antrum	4.30	Par.
4	M	55	6.75	Antrum	3.00	Bif.d.
5	M	46	8.08	Juice	2.69	Par.
				Corpus	4.00	Par.
6	F	44	7.41	Juice	5.30	Bif.d.;Par.;Sca.
				Antrum	4.07	Par.
				Corpus	4.00	Par.
7	F	70	7.83	Juice	4.00	Bif.d.;Par.;Sca.
				Antrum	3.60	Bif.d.
8	M	50	5.02	Antrum	4.00	Bif.ilon.
9	F	43	6.48	Juice	4.00	Un.Bif.

Sca., *S. inopinata*; Par., *P. denticolens*; Bif.d., *B. dentium*; Bif.ilon., *B. infantis-longum*; Un.Bif., Unidentified *Bifidobacteriaceae*

The DNA G + C content of *P. denticolens*, *B. dentium*, and *S. inopinata* was 57±1 mol%, 60±1 mol%, and 46±1 mol%, respectively, consistent with the assigned species (12). Table 1 shows the fermentation characteristics of the isolates in the different substrates assayed. Some phenotypes of the stomach isolates differed from those of the oral cavity. We found that *P. denticolens* fermented melezitose, *S. inopinata* fermented galactose but not salicin, and *B. dentium* did not ferment gluconate: these fermentative characteristics are completely out of line with those of oral cavity isolates of the same species.

All *Bifidobacteriaceae* isolates were nitrate reduction negative.

## Discussion

The study describes the total anaerobic microbial growth, and the presence of *Bifidobacteriaceae* and *Actinomycetales*, in paired gastric biopsies (antrum and corpus) and the gastric juices of OME and AAG patients and normal subjects.

Anaerobic microbial growth was found to be significantly higher in OME and AAG subjects, and absent in normal subjects. There was a significantly higher abundance of *Bifidobacteriaceae* with the species *Bifidobacterium dentium*, *Scardovia inopinata*, and *Parascardovia denticolens* in the OME patients, with respect to the AAG. Furthermore, the distribution of *Actinomycetales* was more homogeneous and was significantly correlated with the OME and AAG subjects, while *Bifidobacteriaceae* presence is significant only for the OME patients.

The strong relationship between high gastric pH and *Bifidobacteriaceae* presence, in greater amounts in OME patients than in AAG, could be due to the fact that the high gastric pH of OME patients can decrease during the day, whereas in AAG patients the pH is constantly high (18). Moreover, in OME, *Bifidobacteriaceae*, which are acidophilic microorganisms, have a higher survival rate because of the varying pH levels. This is the first time that a *Bifidobacteriaceae* population has been studied at the species level in hypochlorhydria stomach: the three species found were *B. dentium*, *P. denticolens*, and *S. inopinata*, typical of the oral cavity (7), with only two isolates belonging to different species, one to *B. infantis-longum* (bifidobacterial species typical of gut microbiota) and the other to an unidentified *Bifidobacteriaceae spp*. The main scope of the work was to assess the presence of bifidobacteria in hypochlorhydria stomach, but, as a serendipitous finding, we also found a relevant presence of bacteria belonging to the *Actinomycetales* order, an important component of oral microbiota (19, 20). The presence of *Bifidobacteriaceae* and *Actinomycetales* supports the hypothesis that a prevalent source of stomach bacteria could be the microbiota of the oral cavity, rather than bacteria from an intestinal habitat (3). Indeed, it is well known that bacteria from the oral cavity can colonize the stomach if the environmental conditions are favorable (21), and such conditions are certainly favorable in the case of hypochlorhydria in AAG and OME patients.

However, previous important studies on gastric hypochlorhydria failed to detect *Bifidobacteriaceae* (3, 8), which is quite surprising. This could have been due to the use of the primer that is mostly used in 16S rRNA sequences; this primer has mismatches with the sequence of *Bifidobacteriaceae* (22, 23).

The same failure concerns *Actinobacteria*, mainly represented by bifidobacteria. In fact, *Actinobacteria* are under-represented in gut phylogenetic descriptions (24), though their active presence has been evidenced (25). The same problem arises in oral microbiota studies: *Bifidobacteriaceae* are routinely isolated from human oral cavity and saliva by classic culture methods, and also by molecular techniques (6, 7), but extensive recent work based on pyrosequencing and microarray assay failed to reveal *Bifidobacteriaceae* in the oral microbioma (26, 27).

Our study shows that *Bifidobacteriaceae* are evidenced if the appropriate methodology is used, especially in OME gastritis patients.

With regard to human health, studies on gut microbiota have gained in importance, with emerging evidence that demonstrates the role of such microbiota in disease (28–32). Of particular interest is the presence of the bifidobacteria, considered probiotic microorganisms useful to the host for their very beneficial activity (33). The exact role of *Bifidobacteriaceae* in gastritis is still to be investigated (34, 35). The results of this study suggest that the healthy stomach does not harbor bifidobacteria, so their presence is not foreseen under normal conditions. Therefore, it is obviously desirable that the gastric microbiota remain at normal values, but altered physiological conditions such as gastritis, *Helicobacter pylori* infection, autoimmune pathology, gastroduodenal ulcers, neoplastic lesions, etc., do not allow this, and microbiota can remain altered for long periods of time (36). This raises the question: Could this atypical overgrowth of gastric microbiota eventually result in a protective or negative role for the host?

One of the answers could deal with the following consideration: in hypochlorhydria, there are reduced levels of gastric juice vitamin C, which is normally secreted by healthy mucosa to inhibit the bacterial synthesis of nitrites to N-nitroso compounds (37), and increased levels of nitrites, the source of carcinogenic N-nitroso compounds (38). Thus, the presence of bifidobacteria in the hypochlorhydria stomach could be beneficial, helping to reduce nitrite concentrations derived from bacterial metabolism and/or from saliva (39) as it modulates the nitrites through acid production by its modulation through acid production (40).

## Conclusions

Our study suggests that *Bifidobacteriaceae* species distributed in the oral cavity habitat can colonize, to quite a large degree, the OME-treated hypochlorhydria stomach. This is a finding that offers a starting point, one requiring further functional studies, to assess the clinical relevance of *Bifidobacteriaceae* in the stomach, and to gain a better understanding of the mechanism underlying *Bifidobacteriaceae* occurrence in OME-treated gastritis. The intriguing question is whether this atypical microbiota colonization exerts positive or negative effect on the host.

## References

[1] Biavati B, Mattarelli P, Goodfellow M, Kampfer P, Busse H-J, Suzuki K-I, Ludwig W, Whitman WB (2012). Genus *Bifidobacterium*. Bergey's manual of systematic bacteriology. The Actinobacteria.

[2] Hunt RH (1988). The protective role of gastric acid. Scand J Gastroenterol.

[3] Bik EM, Eckburg PB, Gill SR, Nelson KE, Purdom EA, Fiz F (2006). Molecular analysis of the bacterial microbiota in the human stomach. PNAS.

[4] Kimura K, Satoh K, Saifuku K, Taniguchi Y, Hiratsuka H (2001). Concept of specification for biopsy sites. Dig Endosc.

[5] Dicksved J, Lindberg M, Rosenquist M, Enroth H, Jansson JK, Engstrand L (2009). Molecular characterization of the stomach microbiota in patients with gastric cancer and in controls. J Med Microbiol.

[6] Beighton D, Gilbert SC, Clark D, Mantzourani M, Al-Haboubi M, Ali F (2008). Isolation and identification of *Bifidobacteriaceae* from human saliva. App Environ Microbiol.

[7] Modesto M, Mattarelli P, Biavati B (2006). Occurrence of the family *Bifidobacteriaceae* in human dental caries and plaque. Caries Res.

[8] Kato S, Nakajima S, Nishino Y, Ozawa K, Minoura T, Konno M (2006). Association between gastric atrophy and *Helicobacter pylori* infection in Japanese children: a retrospective multicenter study. Dig Dis Sci.

[9] Li XX, Wong GL, To KF, Wong VW, Lai LH, Chow DK (2009). Bacterial microbiota profiling in gastritis without *Helicobacter pylori* infection or non-steroidal anti-inflammatory drug use. PLoS One..

[10] Andersson AF, Lindberg M, Jakobsson H, Bäckhed F, Nyrén P, Engstrand L (2008). Comparative analysis of human gut microbiota by barcoded pyrosequencing. PLoS One.

[11] Beerens H (1990). An elective and selective isolation medium for *Bifidobacterium* spp. Lett Appl Microbiol.

[12] Crociani F, Biavati B, Alessandrini A, Chiarini C, Scardovi V (1996). *Bifidobacterium inopinatum* sp. nov. and *Bifidobacterium denticolens* sp. nov., two new species isolated from human dental caries. Int J Syst Bacteriol.

[13] Crociani F, Alessandrini A, Mucci MM, Biavati B (1994). Degradation of complex carbohydrates by *Bifidobacterium* spp. Int J Food Microbiol.

[14] Biavati B, Scardovi V, Moore WEC (1982). Electrophoretic patterns of proteins in the genus *Bifidobacterium* and proposal of four new species. Int J Syst Bacteriol.

[15] Smibert RM, Krieg NR, Gerhart P, Murray RGE, Wood WA, Krieg NR (1994). Phenotypic characterization. Methods for general and molecular bacteriology.

[16] Rossi M, Altomare L, Gonzalez A, Brigidi P, Matteuzzi D (2000). Nucleotide sequence, expression and transcription analysis of the *Bifidobacterium longum* MB219 lacZ gene. Arch Microbiol.

[17] Xia T, Baumgartner JC (2003). Occurrence of *Actinomyces* in infections of endodontic origin. J Endodont.

[18] Martinsen TC, Bergh K, Waldum HL (2005). Gastric juice: a barrier against infectious diseases. Basic Clin Pharmacol Toxicol.

[19] Hall V (2008). *Actinomyces*-gathering evidence of human colonization and infection. Anaerobe.

[20] Kolenbrander PE (2000). Oral microbial communities: biofilms, interactions, and genetic systems. Ann Rev Microbiol.

[21] Yang I, Nell S, Suerbaum S (2013). Survival in hostile territory: the microbiota of the stomach FEMS. Microbiol Rev.

[22] Hayashi H, Sakamoto M, Benno Y (2004). Evaluation of three different forward primers by terminal restriction fragment length polymorphism analysis for determination of fecal *Bifidobacterium* spp. in healthy subjects. Microbiol Immunol.

[23] Frank JA, Reich CI, Sharma S, Weisbaum JS, Wilson BA, Olsen GJ (2008). Critical evaluation of two primers commonly used for amplification of bacterial 16S rRNA genes. Appl Environ Microbiol.

[24] Brooker MR, Dowd V, Camerlengo T, Kumar PS (2011). Target region selection is a critical determinant of community fingerprints generated by 16S pyrosequencing. PLoS One.

[25] Peris-Bondia F, Latorre A, Artacho A, Moya A, D'Auria G (2011). The active human gut microbiota differs from the total microbiota. PLoS One.

[26] Ahn J, Yang L, Paster BJ, Ganly I, Morris L, Pei Z (2011). Oral microbiome profiles: 16S rRNA pyrosequencing and microarray assay comparison. PLoS One.

[27] Ling Z, Kong J, Jia P, Wei C, Wang Y, Pan Z (2010). Analysis of oral microbiota in children with dental caries by PCR-DGGE and barcoded pyrosequencing. Microbial Ecol.

[28] Guinane CM, Cotter PD (2013). Role of the gut microbiota in health and chronic gastrointestinal disease: understanding a hidden metabolic organ. Ther Adv Gastroenterol.

[29] Gareau MG, Sherman PM, Walker WA (2010). Probiotics and the gut microbiota in intestinal health and disease. Nat Rev Gastroenterol Hepatol.

[30] Fukuda S, Toh H, Hase K, Oshima E, Nakanishi Y, Yoshimura K (2011). Bifidobacteria can protect from enteropathogenic infection through production of acetate. Nature.

[31] Imaoka A, Shima T, Kato K, Mizuno S, Uehara T, Matsumoto S (2008). Anti-inflammatory activity of probiotic *Bifidobacterium*: enhancement of IL-10 production in peripheral blood mononuclear cells from ulcerative colitis patients and inhibition of IL-8 secretion in HT-29 cells. World J Gastroenterol.

[32] Nissen L, Pasini L, Biavati B, Malagolini N, Dall olio F, Valle GD (2006). Role of *Bifidobacterium longum* in the induction of apoptotic deletion in the human enterocyte-like Caco-2 cell line. Ann Microbiol.

[33] Gotteland M, Brunser O, Cruchet S (2006). Systematic review: are probiotics useful in controlling gastric colonization by *Helicobacter pylori?*. Aliment Pharmacol Ther.

[34] Gomi A, Harima-Mizusawa N, Shibahara-Sone H, Kano M, Miyazaki K, Ishikawa F (2012). Effect of *Bifidobacterium bifidum* BF-1 on gastric protection and mucin production in an acute gastric injury rat model. J Dairy Sci.

[35] Tuorkey MJ, Abdul-Aziz KK (2012). Molecular pathogenesis of gastric ulcers and strategies for prevention. Res J Pharm Biol Chem Sci.

[36] Naylor G, Axon A (2003). Role of bacterial overgrowth in the stomach as an additional risk factor for gastritis. Can J Gastroenterol.

[37] Schorah CJ, Sobala GM, Sanderson M, Collis N, Primrose JN (1991). Gastric juice ascorbic acid: effects of disease and implications for gastric carcinogenesis. Am J Clin Nutr.

[38] Carboni M, Guadagni S, Pistoia MA, Amicucci G, Lolli D, Palumbo G (1988). Chronic atrophic gastritis and risk of N-nitroso compounds carcinogenesis. Langenbecks Arch Chir.

[39] Ziebarth D, Spiegelhalder B, Bartsch H (1997). N-nitrosation of medicinal drugs catalysed by bacteria from human saliva and gastro-intestinal tract, including *Helicobacter pylori*. Carcinogenesis.

[40] Grill JP, Crociani J, Ballongue J (1995). Effect of bifidobacteria on nitrites and nitrosamines. Lett Appl Microbiol.

